# Is the first urinary albumin/creatinine ratio (ACR) in women with suspected preeclampsia a prognostic factor for maternal and neonatal adverse outcome? A retrospective cohort study

**DOI:** 10.1111/aogs.13123

**Published:** 2017-03-24

**Authors:** Eleni G. Elia, Amy O. Robb, Karla Hemming, Malcolm J. Price, Richard D. Riley, Anna French‐Constant, Fiona C. Denison, Mark D. Kilby, Rachel K. Morris, Sarah J. Stock

**Affiliations:** ^1^ Public Health, Epidemiology and BiostatisticsSchool of Health and Population SciencesCollege of Medical and Dental SciencesUniversity of BirminghamBirminghamUK; ^2^ The Simpson Centre for Reproductive HealthRoyal Infirmary of EdinburghEdinburghUK; ^3^ Primary Care and Health SciencesKeele UniversityNewcastleUK; ^4^ Tommy's Centre for Maternal and Fetal HealthMRC Centre for Reproductive HealthUniversity of Edinburgh Queen's Medical Research InstituteEdinburghUK; ^5^ Birmingham Centre for Women's & Newborn's HealthInstitute of Metabolism and Systems ResearchCollege of Medical & Dental SciencesUniversity of BirminghamBirminghamUK; ^6^ School of Women's and Infant's HealthUniversity of Western AustraliaThe University of Western Australia at King Edward Memorial HospitalCrawleyWAAustralia

**Keywords:** Preeclampsia, cohort study, ACR, albumin to creatinine ratio, risk factors, prognosis, adverse events

## Abstract

**Introduction:**

The aim of this study was to determine the prognostic value of the first urinary albumin/creatinine ratio (ACR) for adverse maternal and neonatal outcomes and how it relates to other prognostic factors.

**Material and methods:**

We performed a retrospective cohort study from December 2009 to February 2012 with analysis of demographic, clinical and biochemical data from two obstetric day assessment units in hospitals in Southeast Scotland. We included 717 pregnant women, with singleton pregnancies after 20 weeks’ gestation, referred for evaluation of suspected preeclampsia and having their first ACR performed. The ability of ACR to predict future outcomes was assessed in both univariable and multivariable logistic regression models. The latter assessed its prognostic value independent of (adjusting for) existing prognostic factors. Primary outcome measures were maternal and neonatal composite adverse outcomes, and a secondary outcome was gestation at delivery.

**Results:**

In all, 204 women (28.5%) experienced a composite adverse maternal outcome and 146 women (20.4%) experienced a composite adverse neonatal outcome. Multivariate analysis of log‐transformed ACR demonstrated that a 1‐unit increase in log ACR is associated with an increased odds of adverse maternal [odds ratio 1.60, 95% confidence interval (CI) 1.45–1.80] and adverse neonatal (odds ratio 1.15, 95% CI 1.02–1.29) composite outcomes, and with reduced gestational age at delivery (coefficient: −0.46, 95% CI −0.54 to −0.38).

**Conclusions:**

ACR is an independent prognostic factor for maternal and neonatal adverse outcomes in suspected preeclampsia. ACR may be useful to inform risk predictions within a prognostic model.

**Abbreviations**
ACR: albumin to creatinine ratio
BMI: body mass index
BP: blood pressure
CI: confidence interval
MAP: mean arterial blood pressure
OR: odds ratio
RR: risk ratio

**Key Message**

Albumin to creatinine ratio is an independent prognostic factor for maternal and neonatal adverse outcomes in suspected preeclampsia, though the prognostic value appears larger for maternal outcomes. Therefore albumin to creatinine ratio could play an important role in healthcare research and clinical practice in the future.

## Introduction

Preeclampsia is defined as the presence of raised blood pressure (BP; ≥140/90 mmHg) after 20 weeks’ gestation, in a previously normotensive non‐proteinuric patient with one or more of the following: significant proteinuria (≥0.3 g/24 h), maternal organ dysfunction or uteroplacental dysfunction 1, 2. Suspected preeclampsia is the most frequent clinical presentation to obstetric units. Preeclampsia is associated with severe complications such as seizures, stroke, multiple organ failure and perinatal mortality if not recognized and managed properly.

The spot urinary protein to creatinine ratio and the albumin to creatinine ratio (ACR) have been studied in patients with renal disease, diabetes and preeclampsia to assess proteinuria. Albumin excretion is considered to reflect glomerular damage more accurately than total protein excretion, and albuminuria may be a marker of systemic endothelial cell dysfunction 3. The majority of international organizations now recommend spot proteinuria tests in the assessment of suspected preeclampsia. ACR has been shown to be an accurate indicator of proteinuria in women with preeclampsia 4, 5, 6. Despite this evidence, the obstetric community has not widely adopted the use of ACR as yet, and protein to creatinine ratio or 24‐h urine collection are more commonly employed.

As well as being useful in the diagnosis of preeclampsia 4, 6, ACR has potential to be useful in predicting adverse pregnancy outcomes 7, 8. New prognostic factors are needed in this area 9, 10, 11, 12. Prognostic factors can guide clinical decision‐making and patient counseling, and inform the design and analysis of new trials 10, 11, 12. They can also improve prognostic models, which produce absolute risk predictions for women based on a set of individual characteristics 9. Before including a new factor in a prognostic model, it is important to quantify its independent prognostic value over and above existing prognostic factors. Factors that add additional (independent) prognostic information are difficult to find, but are necessary to improve the discrimination performance of prognostic models 12.

The aim of this study was to examine the prognostic value of baseline ACR (ACR at first presentation) to predict maternal and neonatal adverse outcomes in women referred with suspected preeclampsia. There were two objectives: (i) to examine whether ACR is prognostic for adverse maternal and neonatal outcomes when no other factor is considered (unadjusted prognostic effect) and (ii) to evaluate whether ACR is a prognostic factor for such outcomes after adjusting for existing prognostic factors (independent prognostic effect).

## Material and methods

### Study design

We performed a retrospective cohort study of pregnant women undergoing ACR test in the obstetric Day Assessment Units of two hospitals in National Health Service Lothian trust between December 2009 and February 2012. The Simpson Centre for Reproductive Health is a tertiary referral center with more than 6500 deliveries per annum. St John's Hospital is a district general hospital with approximately 2600 deliveries per annum. Women were excluded if they had not delivered by the end of February 2012.

All pregnant women with urinary ACR results were identified from a biochemistry database (APEX, ApexHealthware). Women were included if they had booked for their pregnancy prior to 14 weeks and if they were referred from primary care to the hospital Day Assessment Unit with suspected preeclampsia [suspected hypertension (generally ≥140/90 mmHg) and at least 1+ proteinuria on dipstick testing]. Women were excluded if they had multiple pregnancy, proteinuric renal disease or proven urinary tract infection, or if the ACR was measured for another indication (for example diabetes). Women who had their first ACR sent prior to 20 weeks’ gestation were also excluded, as this suggests a chronic hypertensive or proteinuric disorder or underlying renal pathology.

We performed systematic review of medical records collecting predefined characteristics (demographic and clinical) to maximize accuracy and minimize missing data. We used multiple data sources to collect neonatal outcome data in order to increase confidence that no cases of perinatal mortality or significant morbidity were missed. Data were acquired from the maternity electronic patient records database TRAK (supplied by Intersystems) and the neonatal unit electronic patient records database BadgerNet (supplied by Clevermed) systems. Demographic features were recorded at booking visit, clinical and laboratory data at the time of first ACR measurement and subsequent antenatal visits and at delivery, and the outcome of mothers and babies was collected for every pregnancy.

ACR measurement taken on first hospital assessment for suspected preeclampsia was used in the analysis (i.e. follow‐up measurements were not included). ACR was calculated from urine samples in the biochemistry labs of The Royal Infirmary of Edinburgh. Immunoassays (Abbott Architect), turbidmetric and kinetic alkaline picrate (Jaffe) were used to calculate the concentrations of albumin and creatinine, respectively, in the urine sample. From this, the albumin (mg/L)/urine creatinine (mmol/L) was calculated.

Existing prognostic factors were: gestational age at ACR measurement, essential hypertension, preexisting diabetes, gestational diabetes, social deprivation index, body mass index (BMI), mean arterial BP, current smoking status, parity and maternal age recorded from the clinical record at booking (<14 weeks). Deprivation was recorded as social multiple index of deprivation [a postcode‐based Scottish Index of multiple deprivation from 2012 – five groups ranging from most deprived index 1 to least deprived index 5] 13. BMI was recorded as <18.5, 18.5–24.99, 25.0–29.99, 30.0–34.9, 35.0–39.9 and >40. Mean arterial BP(MAP) was recorded as diastolic BP+ 1/3 (systolic BP‐diastolic BP). MAP was used in place of systolic or diastolic BP because previous evidence suggests it is a better prognostic factor for preeclampsia than BP measured during the first or second trimester of pregnancy 14. Data on development of gestational diabetes (to allow exclusion and diagnosed using Scottish Intercollegiate Guidelines network guideline) 15 and gestation at ACR (days) were also recorded.

The primary maternal outcome was a composite adverse maternal outcome, defined as one or more of: use of intravenous magnesium sulfate for seizure prophylaxis, use of intravenous antihypertensives, admission to intensive care unit/or high dependency unit for hypertension, placental abruption, eclampsia or HELLP (haemolysis, elevated liver enzymes, low platelets). The primary neonatal outcome was a composite adverse neonatal outcome, defined as one or more of: iatrogenic preterm delivery <34 weeks, birthweight <5th centile (calculated from sex‐specific birthweight centile charts) 16, abnormal umbilical artery Doppler [absent or reversed end‐diastolic (ARED) flow], arterial cord pH <7.1, need for ventilation, neonatal or intrauterine death. Secondary outcome was gestation at delivery (weeks).

No formal power calculation was performed, and we included all data available over a three‐year time period to maximize sample size. In prognosis research, a typical rule of thumb is that at least 10 events (cases with the outcome of interest) are required to evaluate every one candidate prognostic variable 17. In our study, over 200 women had a maternal composite adverse outcome, thus the sample size was considered adequate for the analysis performed.

In all, 3.9% of women had one or more missing values for data on existing prognostic factors. Due to the small proportion missing we considered a complete case multivariable analysis sufficient 18. Thus, only a complete case analysis was performed, and the relatively few women with missing data were excluded from the multivariable analysis but included in the ACR‐only analysis.

### Primary analyses

The baseline characteristics of the sample were summarized by primary outcome status with differences between groups assessed using unpaired *t*‐tests or Mann–Whitney U‐tests for continuous and chi‐square tests for binary data.

Univariable and multivariable logistic regression models were used to examine the unadjusted and the adjusted (independent) prognostic association of ACR with each binary primary outcome. The multivariable analysis was adjusted for a predefined set of factors that we considered to be prognostic factors, as described above.

For the continuous variable “ACR” the assumption of linearity of the prognostic effect on the log‐odds scale was examined using fractional polynomials. Fractional polynomials of degree two were used to obtain an appropriate transformation for ACR, for which the linearity assumption did not hold 19. This suggested that a logarithmic transformation was needed for ACR. Thus, the logistic models estimated the prognostic value of ACR as summarized by an (adjusted) odds ratio (OR), giving the (adjusted) relative odds of the outcome for two individuals that differ in log‐ACR by 1 unit. To avoid deletion of patients with undefined log‐transformed ACR values [log (0)], 0.01 was added across all the entries of ACR following transformation of the data.

Similarly, univariable and multivariable models were fitted for the secondary outcome, gestation weeks at delivery using linear rather than logistic regression.

For the neonatal composite outcome, gestational age at ACR measurement was adjusted for as a binary outcome after categorizing to age <34 weeks and age ≥34 weeks. This categorization was enforced by the clinical team in advance of the analysis as follows:

- Women who had the first ACR test before 34 weeks represented a group with suspected preterm preeclampsia vs. women with suspected later onset preeclampsia.
- Preterm preeclampsia is a more severe clinical condition and is associated more often with neonatal adverse outcome including premature delivery.
- Part of the composite adverse neonatal outcome is iatrogenic preterm delivery prior to 34 weeks.

The rationale for the above was based on the existing literature 20, 21, 22, 23.

### Secondary analysis

The discrimination performance of the entire multivariable model was summarized to ascertain its potential as a prognostic model, using the apparent C statistic [area under the receiver operating characteristic (ROC) curve] where 0.5 indicates no discrimination (between those with and those without the outcome) beyond chance and 1 indicates perfect discrimination. The C‐statistic is equivalently defined as the probability that the predicted risk for a randomly selected individual with the outcome is higher than that for a randomly selected individual without the outcome 24.

### Sensitivity analysis

Alongside the univariable and multivariable logistic regression analyses to obtain ORs, Poisson regression with robust standard errors was used to obtain (adjusted) risk ratios (RRs). The dataset included extreme values (two entries ACR = 2000 and one entry where ACR = 0). Therefore a sensitivity analysis was run to examine the effect of excluding these values.

All analyses were performed in STATA version 12 (StataCorp, College Station, TX, USA) and the regression models fitted using maximum likelihood estimation.

This was a retrospective study on samples already obtained and the study was approved through the University of Edinburgh and registered with the University of Edinburgh and NHS Lothian on 29/2/2012. No external ethics committee was required. An agreement with the data holder was in place to use the data, for the purposes of this study, which were anonymous and unlinked.

## Results

In all, 941 pregnant women had an ACR performed during the study period. A total of 224 records were excluded due to predefined exclusion criteria, leaving a cohort of 717 women. Complete data (on ACR and existing prognostic factors for the multivariable analysis) was available for 689 women. Women's characteristics are detailed in Table 1. The majority of first ACR measurements were performed between 35 and 40 weeks’ gestation (interquartile range 35–40 weeks, median 37 weeks and standard deviation 4 weeks).

**Table 1:** Baseline maternal characteristics (values are numbers and percentages of the presence of a given characteristic)

Characteristic	Participants (*n* = 717)
Maternal age at delivery (years), mean (SD)	29.93 (6.06)
Booking characteristics	
Nulliparity	57.18%
Essential hypertension	9.34%
Preexisting diabetes	2.79%
Current smoker	15.85%
Scottish index of multiple deprivation	
1 (most deprived)	21.51%
2	22.63%
3	20.39%
4	15.39%
5 (least deprived)	20.11%
Body mass index	
<18.5	2.32%
18.5–24.99	33.48%
25.0–29.99	28.55%
30.0–34.9	20.14%
35.0–39.9	9.71%
>40	5.80%
Booking systolic BP, mean (SD)	115.26 (12.48)
Booking diastolic BP, mean (SD)	69.78 (9.81)
Booking mean arterial BP, mean (SD)	84.94 (9.95)
Development of gestational diabetes	3.35%
Gestational age at ACR test (weeks), median (IQR)	37.43 (35.0–39.14)
ACR result (mg/mmol), median (IQR)	4.40 (1.40–23.60)
Gestational age at delivery (weeks), median (IQR)	39.43 (38.00–40.43)

BP, blood pressure; HDU, high dependency unit; HELLP, hemolysis elevated liver enzymes low platelet count syndrome; ICU, intensive care unit; IQR, interquartile range; SD, standard deviation.

### Adverse maternal outcomes

Of the 717 women included, 204 experienced a composite adverse maternal outcome (28.5%) (Table 2). Thirty women had more than one adverse event (*n* = 174 one event, *n* = 26 two events, *n* = 4 three events), leading to a total of 238 adverse outcomes. Supporting Information Table S1 shows the maternal characteristics for the women with and without composite adverse maternal outcomes. MAP and maternal age at booking were comparable between the two groups. There was no significant difference between the two groups regarding essential hypertension, gestational diabetes, and smoking or social deprivation index. Univariable analysis showed that mean ACR, median gestational age at ACR measurement, mean maternal age, preexisting diabetes and BMI differed between the two outcome groups (Table S1).

**Table 2:** Number of maternal and neonatal adverse outcomes

	Values are numbers
Maternal adverse outcomes (Total *n* = 238)	
Use of magnesium sulfate	12
Use of intravenous antihypertensives	15
Admission to HDU or ICU for hypertension	196
Abruption	7
Eclampsia	0
HELLP	8
Neonatal adverse outcomes (Total *n* = 192)	
Iatrogenic preterm delivery <34 weeks	33
Birthweight <5th centile	98
Abnormal Dopplers (AEDF or REDF)	11
Arterial cord pH <7.1	12
Need for ventilation	32
Intrauterine death	5
Neonatal death	1

AEDF, absent end‐diastolic flow; HDU, high dependency unit; HELLP, hemolysis elevated liver enzymes low platelet count syndrome; ICU, intensive care unit; REDF reversed end‐diastolic flow.

### Adverse neonatal outcomes

Of 717 neonates, 146 experienced a composite adverse neonatal outcome (20.4%) (Table 2). Twenty‐eight neonates had more than one adverse event (*n* = 118 one event, *n* = 15 two events, *n* = 8 three events and *n* = 5 four events), leading to a total of 192 adverse outcomes. Maternal age was comparable between the two groups. There were differences in median gestational age at ACR measurement, mean ACR, smoking, BMI and MAP between the groups (see Supporting Information Table S2).

### Unadjusted and adjusted prognostic value of ACR for maternal and neonatal adverse outcomes

Univariable logistic regression analysis of all 717 women (Table 3) showed that log ACR is prognostic for both maternal [OR 1.52, 95% confidence interval (CI) 1.38–1.684] and neonatal (OR 1.13, 95% CI 1.02–1.25) composite adverse outcome. These unadjusted estimates imply that a unit increase in log‐transformed ACR increases the odds of maternal and neonatal adverse outcomes by 52% and 13%, respectively.

**Table 3:** Logistic regression results for unadjusted and adjusted models for the primary outcomes: composite maternal and composite neonatal outcomes

Model	Variable	Composite maternal adverse outcome			Composite neonatal adverse outcome		
		OR (95% CI)	*p*‐value	ROC^a^	OR (95% CI)	*p*‐value	ROC^a^
Unadjusted	ACR^b^	1.52 (1.38–1.68)	<0.001	0.70 (0.66–0.74)	1.13 (1.02–1.25)	0.022	0.557 (0.504–0.610)
Adjusted	ACR^b^	1.60 (1.42–1.80)	<0.001	0.76 (0.72–0.80)	1.15 (1.02–1.29)	0.025	0.718 (0.668–0.760)
	Gestational age at ACR	0.88 (0.83–0.92)	<0.001		0.25 (0.16–1.29)	<0.001	
	Maternal age	1.04 (1.08–1.08)	0.019		0.99 (0.95–1.02)	0.505	
	Essential hypertension	0.78 (0.38–1.60)	0.505		1.62 (0.79–3.33)	0.19	
	Preexisting diabetes	0.68 (0.12–3.72)	0.655		1.77 (0.40–7.88)	0.452	
	Gestational diabetes	1.02 (0.38–2.77)	0.964		0.73 (0.19–2.77)	0.64	
	Smoking	0.85 (0.50–1.45)	0.55		1.94 (1.16–3.26)	0.012	
	Nulliparity	0.96 (0.66–1.40)	0.826		1.16 (0.77–1.75)	0.471	
	Social deprivation index						
	1	1			1		
	2	0.80 (0.46–1.41)	0.451		0.95 (0.53–1.72)	0.876	
	3	1.10 (0.63–1.92)	0.73		0.78 (0.42–1.45)	0.437	
	4	0.623 (0.33–1.19)	0.152		0.78 (0.39–1.55)	0.473	
	5	0.424 (0.26–0.80)	0.008		0.84 (0.43–1.62)	0.603	
	Body mass index						
	<18.5	1			1		
	18.5–24.99	1.06 (0.32–3.50)	0.93		0.21 (0.07–0.64)	0.006	
	25.0–29.99	1.47 (0.44–4.93)	0.535		0.12 (0.04–0.38)	<0.001	
	30.0–34.9	0.70 (0.20–2.48)	0.581		0.11 (0.03–0.37)	<0.001	
	35.0–39.9	0.50 (0.13–1.98)	0.321		0.16 (0.04–0.57)	0.005	
	>40.0	0.56 (0.13–2.39)	0.434		0.09 (0.02–0.41)	0.002	
	Mean arterial blood pressure	1.02 (1.00–1.05)	0.041		0.99 (0.97–1.01)	0.381	

ROC, receiver operating characteristic.

a C statistic.

b Log‐transformed ACR (albumin creatinine ratio).

Multivariable analysis (based on the 689 women with complete data, Table 3) also showed that log ACR is an independent prognostic factor for maternal composite adverse outcome (OR 1.60, 95% CI 1.43–1.80) and neonatal composite adverse outcome (OR 1.15, 95% CI 1.02–1.29). This implies that a unit increase in log‐transformed ACR, after adjusting for other factors, increases the odds of adverse maternal composite outcome by 60% and of adverse neonatal outcome by 15%.

### Unadjusted and adjusted prognostic value of ACR for gestation at delivery

Univariable (coefficient −0.38, 95% CI −0.48 to −0.27, *p* < 0.001) and multivariable linear regression (coefficient −0.46, 95% CI −0.54 to −0.38, *p* < 0.001) shows a prognostic effect of log ACR for gestational age at delivery (Supporting Information Table S3). The adjusted estimate implies that for every unit increase in log‐transformed ACR, the average gestational age at delivery is decreased by about 0.5 weeks.

### Discrimination performance of the multivariate models

The apparent C‐statistic for the multivariable models was 0.76 (95% CI 0.72–0.80) for composite maternal adverse outcome and 0.72 (95% CI 0.67–0.77) for composite neonatal adverse outcome (Table 3). If ACR is removed, then the C‐statistic of the multivariable models is reduced considerably to 0.67 (95% CI 0.64–0.72) for maternal composite outcome; however, for the neonatal outcome the C‐statistic and its 95% CI barely change. This suggests that ACR is more important in terms of providing additional discrimination as to outcome risk predictions, for the maternal outcome.

### Sensitivity analysis

Results from the Poisson model with robust standard errors were consistent with those of logistic regression analysis. In both the univariable and multivariable analysis ACR still had significant prognostic ability for maternal (unadjusted RR 1.31, 95% CI 1.24–1.39; adjusted RR 1.32 95% CI 1.25–1.41) and neonatal outcomes (RR 1.10, 95% CI 1.01–1.19; adjusted RR 1.10, 95% CI 1.02–1.19) (Supporting Information Table S4). This implies that, after adjusting for other factors, a unit increase in log‐transformed ACR increases the risk of adverse maternal outcome by 32% and of fetal adverse outcome by 10%.

**Figure 1:**
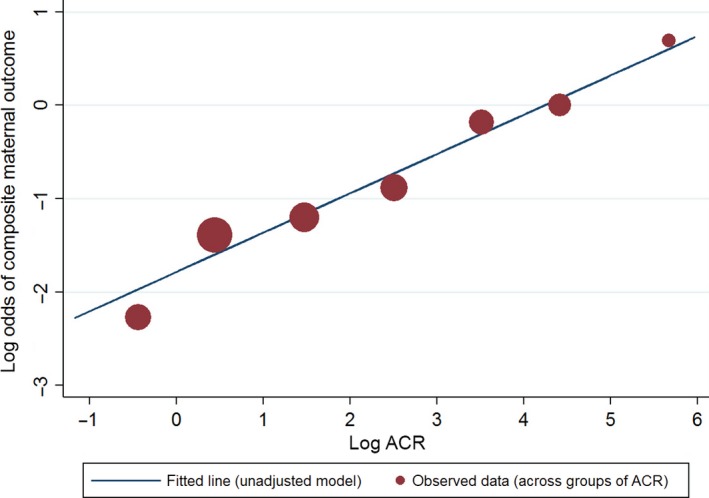
Graph of the predicted probability of maternal composite adverse outcome against albumin (mg/L) creatinine (mmol/L) ratio (ACR). The adjusted (red) and unadjusted (blue) models were fitted using log e (ln)‐transformed ACR and the logit was obtained using the coefficients from the fitted model multiplied by the means/medians of all other continuous adjustment factors, the most common category of the categorical adjustment factors and the values of log ACR. [Color figure can be viewed at wileyonlinelibrary.com]

The sensitivity analysis, excluding the extreme values (ACR = 2000 and ACR = 0), did not alter any conclusions for either primary and secondary outcomes (Supporting Information Tables S5 and S6). Supporting Information Figures S1 and S2 show the predicted probability of maternal adverse composite outcomes for ACR (Figure S1) and log ACR (Figure S2) based on the univariable and multivariable models excluding extreme values (ACR = 2000 and ACR = 0). To illustrate the appropriate fit of a linear relation between log ACR and the log‐odds of a maternal composite outcome, Figure 1 shows the unadjusted linear relationship alongside the observed risk.

## Discussion

Based on this retrospective cohort study, we show that log ACR is an independent prognostic factor for composite adverse maternal and neonatal outcomes. We suggest that a unit increase in log‐transformed ACR is associated with a 30% increased risk of maternal adverse composite outcome and a 10% risk of neonatal adverse composite outcome (corresponding to increased odds of 60 and 15%, respectively). We also demonstrated that in this population a 1‐unit increase in log ACR was associated with a decrease in gestation at delivery by approximately 0.5 weeks (approximately 3 days).

Based on the secondary analyses we showed that although ACR adds prognostic value, the overall discrimination performance of the multivariable models was only moderate. Thus, additional prognostic factors are required to improve performance further, for a clinically useful model to identify those most likely to have an adverse outcome. In terms of improving discrimination performance (as measured by the C‐statistic), ACR appears to be more important for maternal outcomes than for fetal outcomes.

A systematic review 25 and study that used ORs and appropriate tests on two ACR thresholds 26 have already indicated a prognostic ability of ACR for adverse outcomes associated with preeclampsia. Nonetheless, three of five of the studies included in the systematic review 25 were conducted 30 years ago with ACR tests that had different thresholds and that were performed in heterogeneous populations 7. Previous work is also limited by the use of thresholds to categorize (or dichotomize) ACR values 26. Other studies have found that the degree of proteinuria does not correlate with adverse outcome 6, 27. A major strength and uniqueness of our study was that ACR was analyzed as a continuous variable 28. Categorization of continuous predictors leads to loss of information, and hence loss of power, as well as poor predictive performance, and hence poor clinical usefulness 29, 30, 31. It also leads to data dredging (to find the “best” threshold) and does not reflect the underlying prognostic trend.

A log transformation was identified as the most appropriate scale on which to incorporate ACR in the model, suggesting that the effect of a 1‐unit increase in ACR depends on the actual value of ACR itself. Other strengths include the use of stored samples to measure ACR using standardized measurement methods, the collection of ACR values blind to the outcome status, the reasonably large cohort itself, and the very small amount of missing data.

This study had some limitations. The primary outcomes were “composite” to increase the power to detect the prognostic ability of ACR. Moreover, the outcomes are objective, and clinical severity is similar within each group. However, it is difficult to examine the effect size of the prognostic factor of interest for each outcome separately 32. It is instead presumed that the effect size is related to all the components of the composite outcome. It is recommended that components of composite outcomes be considered secondary outcomes and that the related results are provided alongside primary analysis. This was not possible in this study due to the small number of events in most of the components of the composite outcome. However, these components were carefully selected to ensure that they were comparable in magnitude of severity and direction of effect.

A further potential limitation results from the retrospective design of our study, as it is difficult to exclude the possibility of intervention bias in observational studies of this type. ACR results were available to clinicians, and may have influenced management decisions and thereby affected maternal and neonatal outcomes. However, these effects are likely to be small, as decision‐making in women with preeclampsia is based on the whole clinical presentation, not just the amount of proteinuria.

We have shown that in women with suspected preeclampsia the ACR at presentation is an independent predictor of adverse outcome. As an indication of the potential usefulness of ACR in practice, Figure 1 shows how the value of ACR would change the predicted probability of an adverse outcome for a woman who otherwise would have median values of other covariates included in our model. However, clinical management of women with preeclampsia is directed by multiple factors, for example BP control, hematological and biochemical parameters, symptomatology and fetal considerations, including gestation. Thus, no single factor determines management or, in particular, intervention via delivery. Our data suggest that ACR should be considered within this clinical assessment.

A recent series on prognosis research 9, 10, 11, 12 discusses how a single prognostic factor (such as ACR) rarely predicts individual outcome risk accurately, and usually does not suitably discriminate between high‐risk and low‐risk individuals. This is why prognostic models are needed, as they utilize multiple prognostic factors in combination to improve individual risk prediction accuracy and to discriminate better the underlying risk across individuals 33. Future work should focus on identifying further independent prognostic factors for adverse outcomes in order to further improve the discrimination performance of prognostic models. This may include the examination of the prognostic value of multiple measurements of ACR over time. In due course, a prognostic model could be developed incorporating a large set of prognostic factors (including ACR), followed by internal and external validation to ensure reliability of the model predictions. At that stage, its use in clinical decision‐making could be evaluated, for example based on values of high predicted risk that warrant clinical action.

## Funding

There was no funding for this study. S.J.S. and F.D. are supported by Tommy's (registered charity nos 1060508 and SCO39280), who contribute to research infrastructure costs.

## Supporting information


**Table S1.** Maternal characteristics for women who experienced maternal adverse composite outcome; values are numbers and percentages unless otherwise stated.

**Table S2.** Maternal characteristics for neonatals who experienced adverse composite outcomes; values are numbers and percentages unless otherwise stated.

**Table S3.** Linear regression results for the unadjusted and adjusted model for the secondary outcome; gestational age at delivery.

**Table S4.** Poisson regression with robust SE results for ACR (log‐transformed) for unadjusted, adjusted models, where the response is composite maternal/neonatal adverse outcome.

**Table S5.** Logistic regression results with extreme ACR values removed for log‐transformed ACR for unadjusted and adjusted models for the primary outcomes; composite maternal adverse outcome and composite neonatal outcome.

**Table S6.** Linear regression results with extreme ACR values removed for log‐transformed ACR for the unadjusted and adjusted model for the secondary outcome; gestational age at delivery.

**Figure S1.** Graph of the predicted probability of maternal composite adverse outcome (AO) against albumin (mg/L) creatinine (mmol/L) ratio (ACR). The adjusted (red) and unadjusted (blue) models were fitted using log‐transformed ACR and the logit was obtained using the coefficients from the fitted model multiplied by the means/medians of all other continuous adjustment factors, the most common category of the categorical adjustment factors and the values of log ACR.

**Figure S2.** Graph of the predicted probability of maternal composite adverse outcome (AO) against the log‐transformed albumin (mg/L) creatinine (mmol/L) ratio (ACR). The adjusted (red) and unadjusted (blue) models were fitted using log‐transformed ACR and the logit was obtained using the coefficient of log ACR from the fitted model multiplied by the values of log ACR.
