# Supplementary material for: Is the first urinary albumin/creatinine ratio (ACR) in women with suspected preeclampsia a prognostic factor for maternal and neonatal adverse outcome? A retrospective cohort study
Source: Acta Obstet Gynecol Scand. 2017 Mar 24;96(5):580–8. doi: 10.1111/aogs.13123 (PMC5413808; doi:10.1111/aogs.13123)
Supplement: Supplementary file 1 — Table S1. Maternal characteristics for women who experienced maternal adverse composite outcome; values are numbers and percentages unless otherwise stated. [file AOGS-96-580-s001.docx]

Table S1: Maternal characteristics for women who experienced maternal adverse composite outcome, values are numbers and percentages unless otherwise stated

| Characteristic | Maternal no AO  N=513 | Maternal AO  N=204 | p-value |
| --- | --- | --- | --- |
| ACR (mg/mol), median (IQR) | 3.20 (1.20-13.00) | 17.85 (3.20-57.35) | *<*0.001 |
| gestational age at ACR (weeks), median (IQR) | 37.86 (35.86-39.43) | 36.21 (33.57-38.43) | *<*0.001 |
| maternal age (years), mean (SD) | 29.63 (6.16) | 30.69 (5.75) | 0.034 |
| essential hypertension | 47 (9.2%) | 20 (9.8%) | 0.790 |
| pre-existing diabetes | 10 (2.0%) | 10 (4.9%) | 0.030 |
| gestational diabetes | 16 (3.1%) | 8 (3.9%) | 0.590 |
| smoking | 83 (16.2%) | 30 (14.7%) | 0.647 |
| missing | 2 (0.4%) | 2 (1.0%) |  |
| nullparity  social deprivation index: 1 (most deprived) | 291 (56.7%)  105 (20.5%) | 119 (58.3%)  49 (24.0%) | 0.695  0.259 |
| 2 | 117 (22.8%) | 45 (22.1%) |  |
| 3 | 98(19.1%) | 48 (23.5%) |  |
| 4 | 81 (15.8%) | 29 (14.2%) |  |
| 5 (least deprived) | 112 (21.8%) | 32 (15.7%) |  |
| missing body mass index: | . | 1 (0.5%) |  |
| *<*18.5 | 11 (2.1%) | 4 (2.5%) | 0.072 |
| 18.5-24.99 | 163 (31.8%) | 68 (33.3%) |  |
| 25.0-29.99 | 131 (25.5%) | 66 (32.4%) |  |
| 30.0-34.9 | 109 (21.3%) | 30 (14.7%) |  |
| 35.0-39.9 | 55 (10.7%) | 12 (5.9%) |  |
| *>* 40 | 31 (6.0%) | 9 (4.4%) |  |
| missing | 13 (2.53%) | 14 (6.9%) |  |
| mean arterial BP at booking, mean (SD) | 84.78 (9.94) | 85.35 (9.98) | 0.489 |
| systolic BP at booking, mean (SD) | 115.23 (12.37) | 115.34 (12.79) | 0.911 |
| diastolic BP at booking, mean (SD) | 69.55 (9.88) | 70.35 (9.611) | 0.236 |

t test was conducted for continuous variables; Mann-Whitney U test was conducted for skewed continuous variables, and χ2 test for categorical variables. IQR: interquartile range; SD: standard deviation; AO: adverse outcome. All the maternal characteristics were measured at booking except for gestational diabetes and ACR. Gestation at ACR test is also reported in the table.
