# Supplementary material for: Is the first urinary albumin/creatinine ratio (ACR) in women with suspected preeclampsia a prognostic factor for maternal and neonatal adverse outcome? A retrospective cohort study
Source: Acta Obstet Gynecol Scand. 2017 Mar 24;96(5):580–8. doi: 10.1111/aogs.13123 (PMC5413808; doi:10.1111/aogs.13123)
Supplement: Supplementary file 2 — Table S2. Maternal characteristics for neonatals who experienced adverse composite outcomes; values are numbers and percentages unless otherwise stated. [file AOGS-96-580-s002.docx]

Table S2: Maternal characteristics for neonatal who experienced adverse composite outcome, values are numbers and percentages unless otherwise stated

| Characteristic | neonatal no AO  N=571 | neonatal AO  N=146 | p-value |
| --- | --- | --- | --- |
| ACR (mg/mmol), median (IQR) | 3.80 (1.30-22.00) | 6.35 (1.80-33.90) | 0.034 |
| gestational age at ACR (weeks), median (IQR) | 37.71 (35.71-39.29) | 35.71 (32.14-38.29) | *<*0.001 |
| maternal age (years), mean (SD) | 30.04 (6.06) | 29.50 (6.06) | 0.339 |
| essential hypertension | 50 (8.8%) | 17 (11.6%) | 0.285 |
| pre-existing diabetes | 16 (2.8%) | 4 (2.7%) | 0.967 |
| gestational diabetes | 21 (3.7%) | 3 (2.1%) | 0.331 |
| smoking | 78 (13.7%) | 35 (24.0%) | 0.003 |
| missing | 4 (0.7%) | . |  |
| nullparity | 322 (56.39) | 88 (60.27) | 0.398 |
| social deprivation index: |  |  |  |
| 1 (most deprived) | 113 (19.8%) | 41 (28.1%) | 0.180 |
| 2 | 128 (22.4%) | 34 (23.3%) |  |
| 3 | 118 (20.7%) | 28 (19.2%) |  |
| 4 | 91 (15.9%) | 19 (13.0%) |  |
| 5 (least deprived) | 121 (21.2%) | 23 (15.8%) |  |
| missing body mass index: | . | 1 (0.7%) |  |
| *<*18.5 | 6 (1.1%) | 10 (6.9%) | *<*0.001 |
| 18.5-24.99 | 173 (30.3%) | 58 (39.7%) |  |
| 25.0-29.99 | 164 (28.7%) | 33 (22.6%) |  |
| 30.0-34.9 | 119 (20.8%) | 20 (13.7%) |  |
| 35.0-39.9 | 53 (9.3%) | 14 (9.6%) |  |
| *>* 40 | 35 (6.1%) | 5 (3.4%) |  |
| missing | 21 (3.7%) | 6 (4.1%) |  |
| mean arterial BP at booking, mean (SD) | 85.34 (9.82) | 83.39 (10.32) | 0.035 |
| systolic BP at booking, mean (SD) | 115.81 (12.55) | 113.13 (12.01) | 0.021 |
| diastolic BP at booking, mean(SD) | 70.1 (9.55) | 68.51 (10.71) | 0.081 |

t test was conducted for continuous variables; Mann-Whitney U test was conducted for skewed continuous variables, and χ2 test for categorical variables. IQR: interquartile range; SD: standard deviation; AO: adverse outcome. All the maternal characteristics were measured at booking except for gestational diabetes and ACR. Gestation at ACR test is also reported in the table
