# Supplementary material for: Is the first urinary albumin/creatinine ratio (ACR) in women with suspected preeclampsia a prognostic factor for maternal and neonatal adverse outcome? A retrospective cohort study
Source: Acta Obstet Gynecol Scand. 2017 Mar 24;96(5):580–8. doi: 10.1111/aogs.13123 (PMC5413808; doi:10.1111/aogs.13123)
Supplement: Supplementary file 3 — Table S3. Linear regression results for the unadjusted and adjusted model for the secondary outcome; gestational age at delivery. [file AOGS-96-580-s003.docx]

Table S3: Linear regression results for the unadjusted and adjusted model for the secondary outcome; gestational age at delivery

| Model | Variable | Coefficient (95 % CI) | p-value |
| --- | --- | --- | --- |
| unadjusted | ACR∗∗ | -0.377 (-0.480- -0.274 ) | *<*0.001 |
| adjusted | ACR∗∗ | -0.461 (-0.544- -0.378) | *<*0.001 |
|  | gestational age at ACR | 0.406 ( 0.367- 0.446) | *<* 0*.*001 |
|  | maternal age | -0.016 (-0.040-0.010) | 0.212 |
|  | essential hypertension | 0.190 (-0.342-0.723) | 0.483 |
|  | pre-existing diabetes | -0.958 (-2.122-0.206) | 0.106 |
|  | gestational diabetes | -0.773 (-1.548-0.002) | 0.051 |
|  | smoking | 0.100 (-0.286-0.487) | 0.610 |
|  | nullparity | -0.216 (-0.494-0.062) | 0.128 |
|  | social deprivation index: |  |  |
|  | 1 | 1 |  |
|  | 2 | -0.053 (-0.467-0.362) | 0.803 |
|  | 3 | 0.218 (-0.207-0.642) | 0.314 |
|  | 4 | 0.085 (-0.389-0.559) | 0.725 |
|  | 5 | 0.340 (-0.111-0.791) | 0.139 |
|  | body mass index: |  |  |
|  | *<*18.5 | 1 |  |
|  | 18.5-24.99 | 0.359 (-0.580-1.298) | 0.453 |
|  | 25.0-29.99 | 0.551 (-0.401-1.502) | 0.256 |
|  | 30.0-34.9 | 0.527 (-0.452-1.505) | 0.291 |
|  | 35.0-39.9 | 0.854 (-0.182-1.890) | 0.106 |
|  | *>* 40 | 0.799 (-0.311-1.908) | 0.158 |
|  | mean arterial BP | -0.002 (-0.018-.0145) | 0.819 |

** log transformed ACR
