# Supplementary material for: Is the first urinary albumin/creatinine ratio (ACR) in women with suspected preeclampsia a prognostic factor for maternal and neonatal adverse outcome? A retrospective cohort study
Source: Acta Obstet Gynecol Scand. 2017 Mar 24;96(5):580–8. doi: 10.1111/aogs.13123 (PMC5413808; doi:10.1111/aogs.13123)
Supplement: Supplementary file 4 — Table S4. Poisson regression with robust SE results for ACR (log‐transformed) for unadjusted, adjusted models, where the response is composite maternal/neonatal adverse outcome. [file AOGS-96-580-s004.docx]

Table S4: Poisson regression with robust SE results for ACR (log transformed) for unadjusted, adjusted models, where the response is composite maternal/neonatal adverse outcome

| Response | Model | RR (95 % CI) | p-value |
| --- | --- | --- | --- |
| Maternal AO | unadjusted | 1.311(1.236-1.390) | *<*0.001 |
| Maternal AO | adjusted ∗∗ | 1.323(1.245-1.407) | *<*0.001 |
| Neonatal AO | unadjusted | 1.099 (1.014-1.192) | 0.021 |
| Neonatal AO | adjusted ∗∗ | 1.100 (1.021-1.185) | 0.012 |

AO: composite adverse outcome
