# Supplementary material for: Is the first urinary albumin/creatinine ratio (ACR) in women with suspected preeclampsia a prognostic factor for maternal and neonatal adverse outcome? A retrospective cohort study
Source: Acta Obstet Gynecol Scand. 2017 Mar 24;96(5):580–8. doi: 10.1111/aogs.13123 (PMC5413808; doi:10.1111/aogs.13123)
Supplement: Supplementary file 5 — Table S5. Logistic regression results with extreme ACR values removed for log‐transformed ACR for unadjusted and adjusted models for the primary outcomes; composite maternal adverse outcome and composite neonatal outcome. [file AOGS-96-580-s005.docx]

Table S5: Logistic regression results with extreme ACR values removed for log transformed ACR for unadjusted and adjusted models for the primary outcomes: composite maternal adverse outcome and composite neonatal outcome

| Response | Model | OR (95 % CI) | p-value | ROC∗ |
| --- | --- | --- | --- | --- |
| Maternal AO | unadjusted | 1.536 (1.385 1.703) | *<*0.001 | 0.697 (0.654-0.740) |
| Maternal AO | adjusted | 1.615 (1.435 1.818) | *<*0.001 | 0.758 (0.716-0.799) |
| Neonatal AO | unadjusted | 1.139 (1.024 1.266) | 0.017 | 0.558 (0.505-0.611) |
| Neonatal AO | adjusted | 1.158 (1.026 1.307) | 0.018 | 0.717 (0.667-0.768) |

∗C statistic; AO: Composite adverse outcome
