# Supplementary material for: Is the first urinary albumin/creatinine ratio (ACR) in women with suspected preeclampsia a prognostic factor for maternal and neonatal adverse outcome? A retrospective cohort study
Source: Acta Obstet Gynecol Scand. 2017 Mar 24;96(5):580–8. doi: 10.1111/aogs.13123 (PMC5413808; doi:10.1111/aogs.13123)
Supplement: Supplementary file 6 — Table S6. Linear regression results with extreme ACR values removed for log‐transformed ACR for the unadjusted and adjusted model for the secondary outcome; gestational age at delivery. [file AOGS-96-580-s006.docx]

Table S6: Linear regression results with extreme ACR values removed for log transformed ACR for the unadjusted and adjusted model for the secondary outcome; gestational age at delivery

| model | coefficient (95 % CI) | p-value |
| --- | --- | --- |
| unadjusted | -0.378 (-0.483- -0.272 ) | *<*0.001 |
| adjusted | -0.396 (-0.503- -0.885) | *<*0.001 |
