# Supplementary figures and images for: Is the first urinary albumin/creatinine ratio (ACR) in women with suspected preeclampsia a prognostic factor for maternal and neonatal adverse outcome? A retrospective cohort study
Source: Acta Obstet Gynecol Scand. 2017 Mar 24;96(5):580–8. doi: 10.1111/aogs.13123 (PMC5413808; doi:10.1111/aogs.13123)

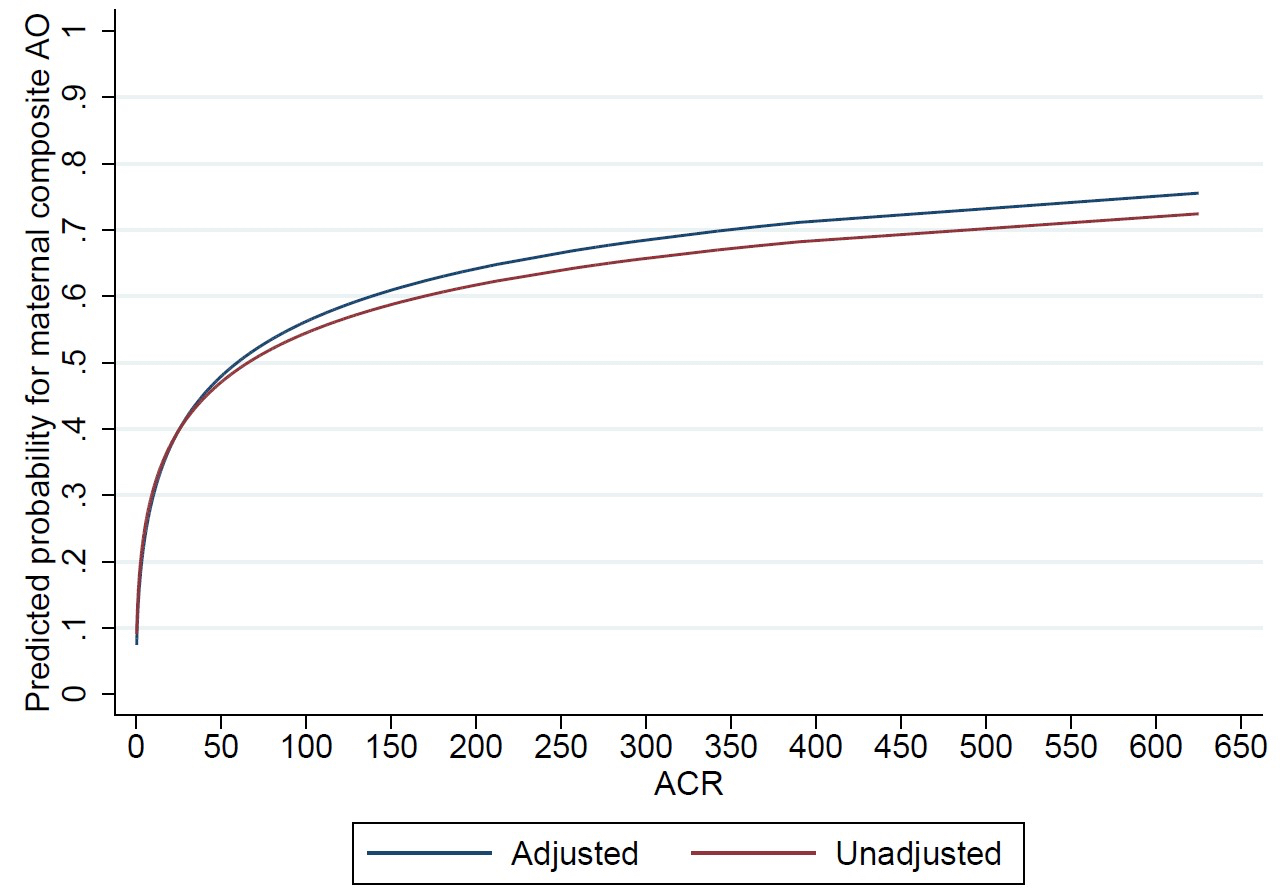

Supplement: Supplementary file 7 — Figure S1. Graph of the predicted probability of maternal composite adverse outcome (AO) against albumin (mg/L) creatinine (mmol/L) ratio (ACR). The adjusted (red) and unadjusted (blue) models were fitted using log‐transformed ACR and the logit was obtained using the coefficients from the fitted model multiplied by the means/medians of all other continuous adjustment factors, the most common category of the categorical adjustment factors and the values of log ACR. [file AOGS-96-580-s007.jpg]

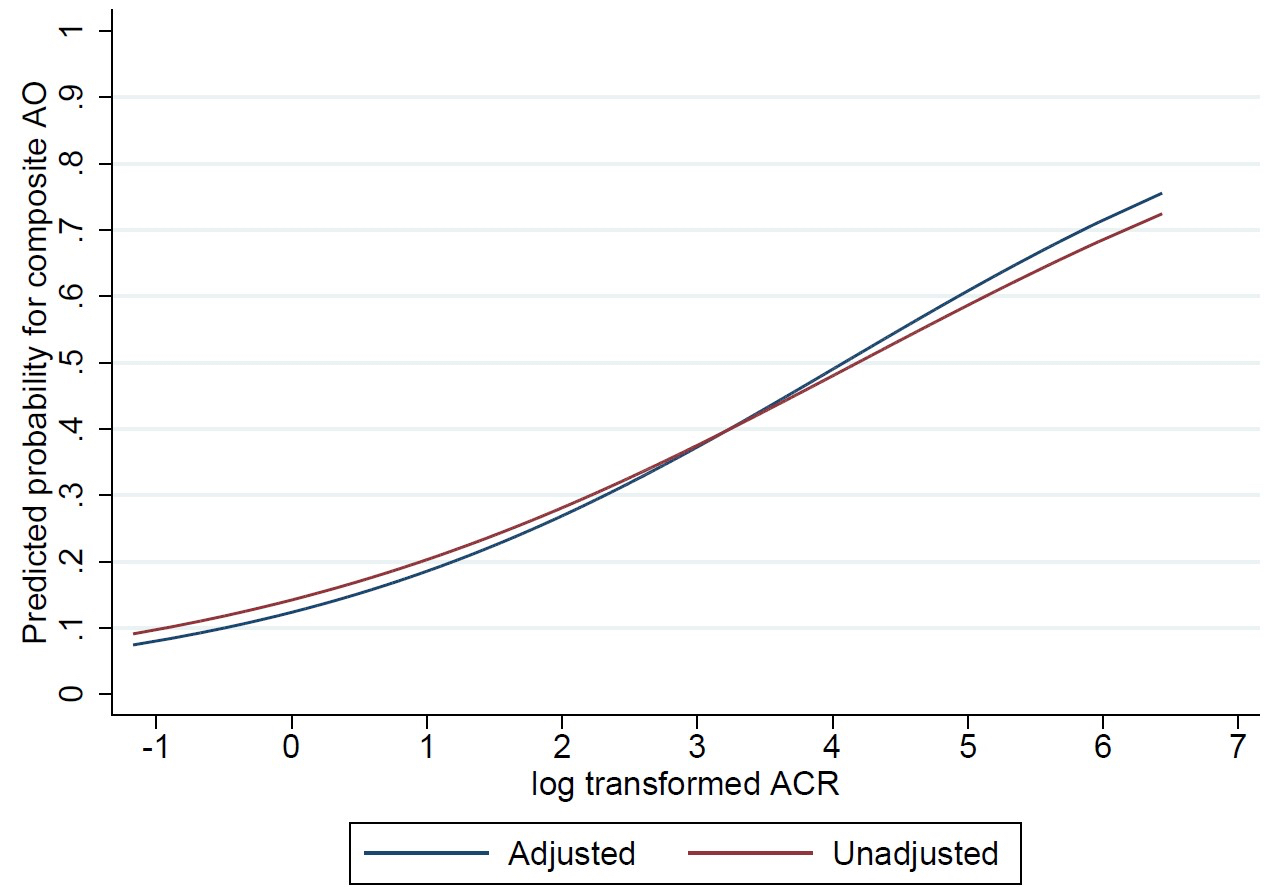

Supplement: Supplementary file 8 — Figure S2. Graph of the predicted probability of maternal composite adverse outcome (AO) against the log‐transformed albumin (mg/L) creatinine (mmol/L) ratio (ACR). The adjusted (red) and unadjusted (blue) models were fitted using log‐transformed ACR and the logit was obtained using the coefficient of log ACR from the fitted model multiplied by the values of log ACR. [file AOGS-96-580-s008.jpg]
